# Perceived weight-related stigma, loneliness, and mental wellbeing during COVID-19 in people with obesity: A cross-sectional study from ten European countries

**DOI:** 10.1038/s41366-022-01220-1

**Published:** 2022-09-14

**Authors:** Rebecca A. Jones, Paul Christiansen, Niamh G. Maloney, Jay J. Duckworth, Siobhan Hugh-Jones, Amy L. Ahern, Rebecca Richards, Adrian Brown, Stuart W. Flint, Eric Robinson, Sheree Bryant, Jason C. G. Halford, Charlotte A. Hardman

**Affiliations:** 1 MRC Epidemiology Unit, University of Cambridge, Cambridge, CB2 0QQ UK; 2 Department of Psychology, University of Liverpool, Liverpool, L69 7ZA UK; 3 School of Psychology, University of Leeds, Leeds, LS2 9JT UK; 4 Centre for Obesity Research, University College London, London, WC1E 6JF UK; 5 Bariatric Centre for Weight Management and Metabolic Surgery, University College London Hospital NHS Trust, London, UK; 6 National Institute of Health Research, UCLH Biomedical Research Centre, London, UK; 7 Scaled Insights, Leeds, LS2 3AA UK; 8 The European Association for the Study of Obesity, Teddington, TW11 8GT UK

**Keywords:** Weight management, Epidemiology

## Abstract

**Background:**

Increased weight-related stigma during the COVID-19 pandemic has amplified the need to minimise the impacts on mental wellbeing. We investigated the relationship between the perceived changes in the representation of obesity in the media and mental wellbeing during the pandemic in a sample of people with obesity across 10 European countries. We also investigated the potential moderating effect of loneliness.

**Methods:**

Between September to December 2020 during the COVID-19 pandemic, participants reported data on demographics, mental wellbeing (measured by World Health Organisation Five Wellbeing Index and Patient Health Questionaire-4), loneliness (measured by De Jong Gierveld short scale), and perceived change in the representation of obesity in media (measured by a study-specific question) using the online, cross-sectional EURopean Obesity PatiEnt pANdemic Survey (EUROPEANS). Data were analysed using linear mixed-effects models, controlling for age, gender, body mass index, and shielding status, with random incept for country.

**Results:**

The survey was completed by 2882 respondents. Most identified as female (56%) and reported their ethnicity as White or White-mix (92%). The total sample had a mean age of 41 years and a BMI of 35.4 kg/m^2^. During the peak of the pandemic, compared to pre-pandemic, perceiving more negative representation of people with obesity on social media was associated with worse psychological distress, depression, and wellbeing. Perceiving more positive representation, compared to no change in representation, of people with obesity on television was associated with greater wellbeing, yet also higher psychological distress and anxiety. Loneliness, as a moderator, explained ≤0.3% of the variance in outcomes in any of the models.

**Conclusions:**

Perceiving negative representation of obesity on social media was associated with poorer mental wellbeing outcomes during the pandemic; positive representation on television was associated with both positive and negative mental wellbeing outcomes. We encourage greater media accountability when representing people with obesity.

## Introduction

Many organisations recognise obesity as a disease, including the American Medical Association, Canadian Obesity Network, World Obesity Federation, and World Health Organisation [1–4]. The European Association for the Study of Obesity (EASO) describes obesity as an adiposity-based chronic disease which is frequent, serious, complex, relapsing, and chronic [5]. Many factors influence the development of obesity, including genetics, gene-lifestyle interactions, and the obesogenic environment [6]. However, societal beliefs and attitudes commonly deem obesity to be due to individual choices and behaviour, resulting in people with obesity often being stereotyped as lazy, greedy, unmotivated, and lacking willpower [7, 8]. These harmful beliefs and attitudes are known as weight-related stigma [9, 10]. Experiences of weight-related stigma are associated with a deterioration in mental wellbeing in people with obesity, including psychological distress, increased social isolation and exclusion, and avoidance of psychologically supportive activities (e.g., healthcare services, physical activity) [7, 9, 11, 12]. Addressing weight-related stigma requires action across multiple levels of influence, including government and policymakers, and is likely to be a long-term process [13, 14]. However, given the short-term impacts of experiencing weight-related stigma, more immediate action is required to mitigate the risk of harm to the mental wellbeing of people with obesity.

Research suggests that the COVID-19 pandemic may have had a negative impact on people with obesity in terms of health-related behaviours and mental wellbeing [15–20]. Multiple factors may have contributed to worsening mental wellbeing in this group, including fear of contracting COVID-19 and potential severity of symptoms, shielding and social isolation, and reduced/no access to services and support [21]. In addition, extensive media coverage and public discourse on television and social media focused on the links between obesity and COVID-19 symptom severity, which many with obesity may have found stigmatising [21, 22]. When covering obesity during the pandemic, the media has been accused of using stigmatising language, promoting a blame culture, and employing tactics to instil both shame and fear in those with obesity [23]. Such a culture of additional stigma and blame is a risk for mental wellbeing in people with obesity, which may occur as a result of internalised blame, shame, and self-stigma [24, 25]. The positioning of media within society – specifically its highly persuasive and influential effect on social norms, beliefs, and attitudes—is thought to play a role in the relationship between media and mental wellbeing in those living with obesity [26–29]. Researchers suggest that the media may exacerbate weight stigma through reinforcing the idea of the ‘ideal’ body shape or size, resulting in the devaluation of those with overweight or obesity and, consequentially, reduced mental wellbeing of these individuals. For example, the media often portrays a person with a thin or muscular physique receiving positive reinforcement and feedback, whilst a person with overweight or obesity is ridiculed and demeaned via negative and degrading comments and weight-related “humour” [26–29].

Therefore, given the short-term adverse impacts of experiencing weight-related stigma, more immediate action is required to mitigate the risk of harm to the mental wellbeing of people with obesity. Some factors, such as loneliness (or lack thereof), may influence the effect of weight-related stigma on the mental wellbeing of people with obesity. The introduction of COVID-19 restrictions (e.g., stay at home orders and social distancing guidelines) reduced individuals’ access to their usual support networks [18–20], with recent research showing that feelings of loneliness were substantially higher during the pandemic [30–33]. Loneliness has been described as “a subjective, negative feeling related to the deficient social relations” and “a feeling of disconnectedness or isolation” [34]. De Jong proposed that loneliness is multidimensional, including emotional (i.e., absence of an intimate relationship) and social loneliness (i.e., absence of a broader social network) [35, 36]. Greater feelings of loneliness may reduce a persons’ ability to regulate emotions and manage the impact of a distressing event, such as weight-related stigma, on their mental wellbeing [37–39]. Alternatively, lower feelings of loneliness may be protective of mental wellbeing in response to experiences such as weight-related stigma. Loneliness during the pandemic may have been particularly important to consider when explaining poor mental wellbeing among individuals living with obesity as, for example, in the United Kingdom many people with more complex obesity were advised to shield and to socially isolate from other members of the population [40].

We investigated the relationship between the perceived representation of obesity in the media (as an indicator of perceived experiences of weight-related stigma) and mental wellbeing during the COVID-19 pandemic in a sample of people with obesity across 10 European countries. We proposed that loneliness may moderate the relationship between perceived experiences of weight-related stigma and mental wellbeing. Specifically, we anticipated that the relationship between perceived experiences of weight-related stigma and poor mental wellbeing (compared to good mental wellbeing) would be most pronounced in people with higher as opposed to lower levels of loneliness. We viewed mental wellbeing as a broad spectrum spanning poor mental health and disorders (e.g., low mood, clinical depression, anxiety) through to positive mental wellbeing. Defining mental wellbeing in this way is associated with reduced stigma, and enables the investigation of a broader range of mental wellbeing outcomes [41–43].

## Methods

### Data source

EURopean Obesity PatiEnt pANdemic Survey (EUROPEANS) was a large international consortium study commissioned to understand the lived experience of obesity during the COVID-19 pandemic. Data from the EUROPEANS study were collected from September 3rd to December 19th 2020. The survey was created using Qualtrics^TM^ (a commercial survey sampling and administrative company). Participants were recruited using several methods: (1) through Qualtrics^TM^ using their existing participant recruitment panels, (2) inviting individuals who had previously opted in to be contacted for research studies (i.e., notified through emails, applications & text message notifications), and (3) via several patient-led obesity advocacy organisations (e.g., European Collaboration for People with obesity (ECPO)). Participants were eligible to take part in the study if they had the following characteristics: self-reported body mass index [BMI] ≥ 30 kg/m^2^, aged ≥18 years, and a resident of one of the specified 10 European countries. We also stratified recruitment by age in years (18–30, 30–50, 50+). Participants were invited to take part and opted in by activating the EUROPEANS survey link which directied them to the study information, consent pages, and survey instrument. Ineligible respondents were immediately exited from the survey after providing a response that did not meet the eligibility criteria or exceeded set quotas (e.g., did not provide consent, BMI was outside the threshold). The survey was completed directly on the Qualtrics^TM^ portal and participants received an honorarium (e.g., reimbursement for their time via cash, gift cards, redeemable points, vouchers).

To ensure data quality, responses were checked for (i) speeding (i.e., respondents with a survey duration of ≤ one-third of the median duration of the survey) and (ii) poor quality [e.g., selecting the first option for every question, bad verbatims (answering open-end questions by way of keyboard banging or typing illogical gibberish answers), duplicate responses and bots (software created to take surveys multiple times for incentives), incomplete data sets] (see Supplementary Materials for greater detail). Respondents who failed any of these quality checks were excluded from the final sample. The study was approved by the University of Liverpool Research Ethics Committee (ref no: 7932 - EUROPEANS study). Written informed consent was obtained electronically from each participant.

### Participant eligibility

Upon clicking on the EUROPEANS survey link, individuals provided informed consent and were asked to complete a self-screening tool to assess their eligibility. Eligibility criteria were: people who were living with obesity during COVID-19 who had a self-reported BMI ≥ 30 kg/m^2^, aged 18 years and above, and were a resident of one of the following countries at the time of completing the survey: England, Greece, Germany, France, Sweden, Israel, Portugal, Italy, Denmark and Spain. The number of participants recruited from each country ranged from 276 to 297.

### Outcomes

The survey collected self-reported demographic information from participants. Pertinent to this study, participants self-reported gender, ethnicity, BMI, and shielding status. The survey collected information about weight management, exposure to media, perceived change in the representation of obesity in media, health behaviours, emotional wellbeing, food security, and COVID-19 exposure. In the following sections, we detail the outcomes relevant to the present study. Prefer not to say options were included for all questions.

#### Perceived changes in the representation of people with obesity in the media

Participants self-reported whether they perceived a change in the representation of people with obesity during the self-defined peak exposure of the outbreak compared to before the COVID-19 pandemic. Participants reported whether they perceived more positive representation, more negative representation, or no change in representation on (i) television and (ii) social media (See Supplementary Materials for specific question wording). Self-reported perceived changes in the representation of people with obesity in the media was used as an indicator of perceived experiences of weight stigma (i.e., perceiving more negative change in media representation was interpreted as experiencing a higher level of weight-related stigma).

#### Depression and anxiety

The Patient Health Questionnaire-4 (PHQ-4) was used as a brief measure of depression and anxiety symptoms over the previous 2 weeks [44]. The PHQ-4 is a 4-item, 4-point self-report Likert scale consisting of 2-items for depression and 2-items for anxiety. Higher PHQ-4 scores indicate a greater burden of depression and anxiety symptoms. It is important to note that the PHQ-4 is not diagnostic, yet can be an indicator of symptom burden [44, 45]. Evidence supports the reliability and validity of this very brief scale to measure depression and anxiety symptoms [44, 45]. In the current sample, the full scale had good reliability (McDonald’s omega (ω) = 0.91). As both subscales consist of two items, reliability was calculated using the Spearman–Browne coefficient [46]; both had good reliability (anxiety r_sb_ = 0_._87, depression r_sb_ = 0_._82).

#### Psychological wellbeing

The World Health Organisation Five Wellbeing Index (WHO-5) is a short self-report measure of subjective psychological wellbeing over the previous 2 weeks [47]. It consists of five items on a 5-point Likert scale, with higher scores representing better psychological wellbeing. Evidence from a systematic review supports the validity of the measure and reports that the scale has been used successfully across a wide range of study fields [47]. In the current sample, internal reliability was good with McDonald’s omega (ω) of 0.93 [48].

#### Loneliness

The De Jong Gierveld (DJG) short scale is a 6-item measure of overall, emotional, and social loneliness. Emotional loneliness relates to the absence of an intimate relationship, whilst social loneliness refers to the absence of a broader social network [35, 36]. The items are answered on a 5-point Likert scale about respondents’ experiences over the previous 2 weeks. Higher scores on the De Jong Gierveld short-scale represent a greater degree of loneliness. Previous research has shown the measure to be a reliable and valid scale [35, 36]. In the current sample, internal reliability was good with ω of 0.87.

### Statistical analysis

Statistical analyses were performed using R version 4.0.5 (Vienna, Austria). Data were analysed using linear mixed-effects models, with total distress (total PHQ score with subscales for depression and anxiety symptoms) or wellbeing as outcome variables. Perceived change in the representation of obesity on social media and TV (positive change/no change/negative change) were used as explanatory variables, whilst age (years), gender (male vs. female), BMI (kg/m^2^), shielding (yes vs. no), socioeconomic status (education status), ethnicity (White vs. BAME), and total loneliness (total score) were control variables. In all models, we added a random intercept for country which significantly improved the fit compared to the models without random effects for all outcomes (Χ^2^ (1) >19.88, *p* < 0.001, Akaike’s Information Criterion (AIC) differences >17).

We ran the models with the control variables and then compared the fit of these control models to those with perceived change in the representation of obesity on social media and television added; in all models, the inclusion of the perceived change in representation variables facilitated better fit (Table 1). The two perceived change in representation variables (i.e., social media and television) were added as a fixed effect with “no change” used as the reference group. Residuals had a normal distribution and there was no evidence of multicollinearity (all variance inflation factors (VIFs) <2.89). As there was some evidence for a small degree of heteroscedasticity in the total distress (total PHQ score) models, we re-ran all models with robust standard errors (RSE). The pattern of results was identical (see Tables S1–S3 in Supplementary Materials for models with RSE).

**Table 1:** Comparison of fit between control and full model.

Outcome	Control AIC	Full model AIC	X^2^ difference
Total distress (PHQ)	14421	14372	56.05*
Anxiety symptoms (PHQ subscale)	10986	10952	42.51*
Depression symptoms (PHQ subscale)	10913	10866	54.64*
Wellbeing (WHO-5)	25559	25519	47.07*

Χ^2^ df = 4

*AIC* Akaike’s Information Criterion, *PHQ-4* Patient Health Questionnaire-4, *WHO-5* World Health Organisation Five Wellbeing Index

*p < 0.001

To explore the effect of loneliness as a moderator of the impact of perceived representation on distress, we computed interaction terms between loneliness (mean-centred) and dummy-coded “more negative” and ‘more positive representation’ for both television and social media. These were added to the regression models described in “Association of changes in the perceived representation of people with obesity on television before and during COVID-19 with mental wellbeing” and “Association of perceived changes in the representation of people with obesity on social media before and during COVID-19 with mental wellbeing”.

## Results

### Participant characteristics

In total, 2882 adults with obesity completed the EUROPEANS survey. Fifty-six percent of survey respondents identified as female, and the total sample had a mean age of 40.6 years and BMI of 35.4 kg/m^2^. The majority of survey respondents reported their ethnicity as White or a White-mix (92%). Table 2 presents further details of participant characteristics. Participant characteristics for key study outcomes by country of residence are presented in Table S4 in the Supplementary Materials.

**Table 2:** Characteristics of the study participants.

Characteristic (*n* = 2882)		Number of participants and percentage (unless specified)
Age (years) [Mean ± standard deviation]		40.6 ± 15.1
Body mass index (kg/m^2^) [Mean ± standard deviation]		35.4 ± 5.9
Gender	Male	1274 (44%)
	Female	1608 (56%)
Ethnicity	White or White Mixed	2661 (92%)
	Asian	58 (2%)
	Black	23 (1%)
	Hispanic or Latino	94 (3%)
	Any other ethnic group	15 (1%)
	Not stated	31 (1%)
Marital status	Common-law partner/ Married/ Civil partnership	1518 (53%)
	Widowed, divorced, or separated (after marriage or civil partnership)	317 (11%)
	Single (never married)	1009 (35%)
	Other	38 (1%)
Educational attainment	Secondary/high school or lower	892 (31%)
	Secondary/higher vocational school	791 (27%)
	University degree or higher	1150 (40%)
	Not stated	49 (2%)
Self-reported shielding in the previous 2 weeks	Yes	1078 (37%)
	No	1804 (63%)

### Association of changes in the perceived representation of people with obesity on television before and during COVID-19 with mental wellbeing

Compared to before COVID-19, perceiving more negative representation of people with obesity on television during peak exposure of the outbreak was not associated with depression symptoms (*B* 0.09, 95% CI: −0.13, 0.31), anxiety symptoms (*B* 0.19, 95% CI: −0.04, 0.41), or wellbeing (*B* 0.99, 95% CI: −1.79, 3.78) when compared to perceiving no change in representation (Table 3). Conversely, compared to before COVID-19, perceiving more positive representation of people with obesity on television during peak exposure of the outbreak was associated with higher levels of anxiety symptoms (*B* 0.26; 95% CI: 0.06, 0.46) and wellbeing (*B* 4.52; 95% CI: 2.01, 7.02) when compared to perceiving no change in representation (Table 3).

**Table 3:** Associations between explanatory variables and depression symptoms, anxiety symptoms, and wellbeing (controlled for age, shielding, body mass index, total loneliness, and gender).

Perceived representation of people with larger bodies during peak exposure of the outbreak, compared to before COVID-19		Estimated association with the outcome when compared to no change (Unstandardised coefficient, SE (95% CI) *p*-value)		
		Depression symptoms	Anxiety symptoms	Wellbeing
Perceived representation on television (‘no change’ as base)	More negative representation	0.09, SE = 0.11 (−0.13 to 0.31) *p* = 0.429	0.19, SE = 0.11 (−0.04 to 0.41) *p* = 0.100	0.99, SE = 1.42 (−1.79 to 3.78) *p* = 0.485
	More positive representation	0.19, SE = 0.10 (−0.01 to 0.39) *p* = 0.054	0.26, SE = 0.10 (0.06 to 0.46) *p* = 0.011	4.52, SE = 1.28 (2.01 to 7.02) *p* < 0.001
Perceived representation on social media (‘no change’ as base)	More negative representation	0.50, SE = 0.11 (0.29 to 0.71) *p* < 0.001	0.31, SE = 0.11 (0.10 to 0.52) *p* = 0.004	−3.52, SE = 1.36 (−6.19 to −0.86) *p* = 0.010
	More positive representation	0.17, SE = 0.10 (−0.02 to 0.37) *p* = 0.080	0.17, SE = 0.10 (−0.03 to 0.36) *p* = 0.093	1.99, SE = 1.25 (−0.47 to 4.45) *p* = 0.112
Proportion of total variance (Conditional R^2^)		0.28	0.25	0.30

*SE* Standard Error

When compared to before COVID-19, perceiving more negative representation of people with obesity on television during peak exposure of the outbreak was not associated with total distress when compared to perceiving no change in representation (*B* 0.27, 95% CI: −0.13, 0.68) (Table S5 in Supplementary materials). Conversely, compared to before COVID-19, perceiving more positive representation of people with obesity on television during peak exposure of the outbreak was associated with higher levels of total distress (*B* 0.45; 95% CI: 0.09, 0.81) when compared to perceiving no change in representation (Table S5 in Supplementary Materials). Tables S6–8 in the Supplementary Materials present the effect of the control variables on the models.

### Association of perceived changes in the representation of people with obesity on social media before and during COVID-19 with mental wellbeing

Compared to before COVID-19, perceiving more negative representation of people with obesity on social media during peak exposure of the outbreak was associated with higher levels of depression (*B* 0.50; 95% CI: 0.29, 0.71) and anxiety symptoms (*B* 0.31; 95% CI: 0.10, 0.52), and lower levels of wellbeing (*B* −3.52; 95% CI: −6.19, −0.86) when compared to perceiving no change in representation (Table 3). Conversely, compared to before COVID-19, perceiving more positive representation of people with obesity on social media during peak exposure of the outbreak was not associated with changes in depression symptoms, anxiety symptoms, or wellbeing when compared to perceiving no change in representation (Table 3).

When compared to before COVID-19, perceiving more negative representation of people with obesity on social media during peak exposure of the outbreak was associated with higher levels of total distress (*B* 0.8; 95% CI: 0.42, 1.19) when compared to perceiving no change in representation (Table S5 in Supplementary materials). In contrast, when compared to before COVID-19, perceiving more positive representation of people with obesity on social media during peak exposure of the outbreak was not associated with total distress (*B* 0.34; 95% CI: −0.02, 0.70) when compared to perceiving no change in representation (Table S5 in Supplementary materials). Tables S6–8 in the Supplementary Materials present the effect of the control variables on the models.

### The moderating effect of loneliness on the association between the representation of people with obesity and mental wellbeing

There was limited evidence to suggest that loneliness moderated the impact of perceived experiences of weight-related stigma on mental wellbeing. For depression symptoms, the moderation model was a marginally better fit (Χ^2^ (4) = 10.92, *p* = 0.028), although it is notable the AIC difference was minor (<4; moderation model AIC = 10864, previous model AIC = 10867), with the R^2^ difference also being minimal (R^2^ change = 0.003). This is due to the significant (*p* = 0.024) moderating effect of loneliness on the association between the positive representation of people with obesity on social media and depression scores (Table 4). Simple slopes suggest that the association between more positive social media representation and higher depression symptoms was significant when loneliness was lower (−1SD, *B* 0.41, SE = 0.14; 95% CI: 0.13 to 0.70), but not at mean or +1 SD. For anxiety, there was no difference between the moderation model (AIC = 10952) and the previous model (AIC = 10948, Χ^2^ (4) = 4.06 *p* = 0.397), with the R^2^ difference being minimal (R^2^ change <0.001). Likewise, there was no difference between the wellbeing model (AIC = 25520) and the previous model (AIC = 25521, Χ^2^ (4) = 8.61, *p* = 0.072), with the R^2^ difference being minimal (R^2^ change = 0.002). Overall, we found limited evidence to suggest that loneliness moderated the relationship between representation of people with obesity of TV or social media and mental wellbeing, with the addition of loneliness as a moderator explaining less than ≤0.3% of the variance in outcomes in any of the three models.

**Table 4:** Moderating effect of loneliness on the associations between explanatory variables and depression, anxiety, and wellbeing (controlled from age, shielding, body mass index, total loneliness, and gender).

Perceived representation of people with obesity during peak exposure of the outbreak, compared to before COVID-19 by loneliness score	Estimated association with the outcome when compared to no change (Unstandardised coefficient, SE (95% CI))		
	Depression symptoms	Anxiety symptoms	Wellbeing
Perceived more negative television representation x loneliness score	0.08, SE = 0.06 (−0.04, 0.21) *p* = 0.193	−0.01, SE = 0.07 (−0.14, 0.12) *p* = 0.874	0.39, SE = 0.82 (−1.22, 2.00) *p* = 0.636
Perceived more positive television representation x loneliness score	0.02, SE = 0.06 (−0.10, 0.13) *p* = 0.780	0.01, SE = 0.06 (−0.11, 0.12) *p* = 0.897	0.53, SE = 0.73 (−0.90, 1.97) *p* = 0.466
Perceived more negative social media representation x loneliness score	<0.01, SE = 0.06 (−0.12 to 0.12) *p* = 0.983	<0.01, SE = 0.06 (−0.12, 0.12) *p* = 0.950	−0.14, SE = 0.77 (−1.64, 1.37) *p* = 0.858
Perceived more positive social media representation x loneliness score	−0.13, SE = 0.06 (−0.23, −0.02) *p* = 0.024	−0.09, SE = 0.06 (−0.20, 0.02) *p* = 0.101	1.30, SE = 0.70 (−0.08, 2.68) *p* = 0.065
Proportion of total variance (Conditional R^2^)	0.28	0.25	0.30

*SE* Standard Error

## Discussion

We investigated the relationship between the perceived representation of obesity on television and social media and mental wellbeing during the COVID-19 pandemic in a sample of people with obesity across 10 European countries. We found that perceiving more negative representation of people with obesity on social media during peak exposure of the pandemic was associated with higher levels of psychological distress and depression symptoms as well as lower levels of wellbeing. During the peak of the COVID-19 pandemic, compared to pre-pandemic, perceiving more positive representation of people with obesity on television was associated with greater symptoms of psychological distress and anxiety, and also higher levels of wellbeing. We also found marginal evidence to suggest that there was a stronger association between the positive representation of obesity on social media and greater depression symptoms when loneliness was lower; However, this association was small in size and not consistent across study findings. Due to the minimal effect and inconsistency across findings, we deemed it inappropriate to base implications or conclusions on this.

A recent systematic review of 105 studies conducted before the COVID-19 pandemic reported that experiencing weight-related stigma (i.e., negative representation of people with obesity) was associated with low mental wellbeing, including poor quality of life, depressive symptoms, and dissatisfaction with body image [7]. This finding aligns with the review of recent research by Ata and colleagues – in this review, the authors concluded that the stigmatising negative portrayals of obesity in wide ranging medias (e.g., television and social media) can be linked to psychological harm for both viewers and those with obesity [26]. Similarly, we found that perceiving more negative representation on social media during the COVID-19 pandemic compared to pre-pandemic was associated with reduced mental wellbeing. However, we also found that perceiving increased negative representation on television appeared to have no impact on mental wellbeing. This may be explained by the differences in how we experience television and social media [27, 49–52]. Clark and colleagues describe the weight stigma impacts of interacting with these medias as structural (i.e., beliefs encoded into society through consumed media), interpersonal (i.e., person-to-person interactions), and intrapersonal (i.e., internalised bias) [27]. Television is suggested to be associated with structured stigma as it is viewed but not interacted with, whereas social media contributes to structured, inter-, and intra-personal stigma as it is both viewed and interacted with via, for example, publicly posted comments [27]. The difference in how we interact with each type of media may contribute to explaining the differences by platform [26, 27]. For example, it is possible that viewing a television advert representing adults with obesity as “lazy” or “greedy” may have a lesser negative impact on mental wellbeing than viewing the same advert on social media alongside potentially stigmatising comments posted by the public. Furthermore, there are several regulations covering the content of television media (e.g., Ofcom Broadcasting Code) [53], whilst social media remains unregulated in comparison; this may further explain the differences by platform. Future research may wish to further investigate why the impact of stigma appears to differ by media platform to better understand the potential reasons for these findings.

Interestingly, we found that experiencing more positive representation of people with obesity on television during the pandemic, compared to before the pandemic, was associated with better psychological wellbeing, yet also higher psychological distress and anxiety. This may be due to the increased media reporting on (i) obesity as a risk factor for more severe COVID-19 complications and death and (ii) the introduction of a new obesity strategies and policies aiming to support those living with obesity (e.g., the new Obesity Strategy in the UK) [21, 23, 54]. Television reporting on the association between COVID-19 and obesity may have increased distress and anxiety; these reports may have reduced an individuals’ sense of perceived control over their weight and health, contributing to increased feelings of worry, anxiety, and concern. However, alongside this, reporting of the new strategies may have increased feelings of psychological wellbeing through feeling supported.

Addressing weight-related stigma requires action from multiple levels of influence and is likely to be a long-term process [13], and introducing relevant policies for media organisations would be a step forward in this process. We recommend and encourage greater media accountability when representing people with obesity, such as the introduction of legislation and regulations to avoid negative portrayals and stigmatising representation. Greater media accountability may also include highlighting the non-individual determinants of obesity (e.g., environmental, marketing) and using accurate, non-biased, respectful images which may be sourced, for example, from the EASO Obesity Image Bank or the World Obesity Image Bank [55, 56]. The use of non-biased and respectful images is noted to reduce weight-related stigma in the media [56].

We proposed that the relationship between perceiving more negative representation of obesity on television and social media and lower well-being may be particularly pronounced among participants with higher levels of loneliness. We found limited and inconsistent evidence suggesting that feelings of loneliness moderated the association between perceived representation on media and mental wellbeing. A recent systematic review, conducted before the COVID-19 pandemic, found that social support did not moderate the relationship between weight-related stigma and mental wellbeing, aligning with the findings of this study [7]. Social support and loneliness are strongly correlated, yet it is possible to have good social support and experience loneliness [7, 34]. Authors of the systematic review identified that there was a considerable lack of research assessing the moderating effects of social support on the relationship between weight-related stigma and mental wellbeing; similar can be noted for the evidence base assessing the moderating effect of loneliness. Future research should aim to investigate the influence of loneliness and social support separately on the impact of weight-related stigma to strengthen the evidence base, as well as investigating various other potential moderators (e.g., history of psychological treatment could protect mental wellbeing).

### Strengths and limitations

This study is strengthened by the large sample of participants across 10 European countries, thus supporting the generalisability of the study findings. Whilst experiences of key study outcomes (perceived representation of obesity in media, mental wellbeing, and loneliness) differed across countries (see supplementary materials), the potential confounding effect of this was controlled for in statistical analyses, mitigating the risk of bias. Relevant control variables were included in statistical analyses to minimise the potential impact of confounders, reducing the likelihood of drawing inaccurate conclusions from the analyses. However, we were restricted by the variables available to control for. For example, we included highest educational attainment to control for the potential confounding effect of socioeconomic status (SES) and acknowledge that alternative measures (e.g., Index of Multiple Deprivation) may represent SES more accurately. Education status is one of the most commonly used measures of SES and is recognised as a suitable measure of SES [57].

We assessed changes in perceived representations of obesity in media (i.e., as an indicator of perceived stigmatising experiences) based on data collected at one-time point, often asking participants to compare their experiences during the peak of the pandemic to pre-pandemic. Consideration should be taken of this when interpreting findings due to the retrospective, cross-sectional and subjective nature of this measure. As the situation relating to the pandemic differed within and between countries, the phrasing of the “peak” of the pandemic allowed participants to self-define when the context of pandemic was most heightened; consideration of this should be taken when interpreting the findings. The measure used was study-specific due to the lack of a COVID-19 specific validated measure of changes in the perceived representation of obesity in media. Future research should consider using mixed methods longitudinal research designs to quantify the relationships between the key factors as well as exploring the thoughts, feelings, and meanings underpinning these findings.

Additionally, reverse causality may in part explain the observed findings as poorer mental wellbeing is associated with distorted interpretations [58–61]. For example, it is possible that those experiencing poor mental wellbeing during the pandemic may have perceived greater negative representations of obesity where there were none, or may be more likely to focus on any true negative representations they observed. Finally, it should be noted that the ethnicity of the participant population was 92% White or White mixed, therefore this should be considered when interpreting the data and determining generalisability of the findings to other situations or contexts.

## Conclusion

During the peak of the COVID-19 pandemic, compared to pre-pandemic, perceiving more positive representation of people with obesity on television with associated with greater symptoms of total distress and anxiety, and greater wellbeing among adults living with obesity. Conversely, perceiving more negative representation of people with obesity on social media was associated with higher levels of total distress and depression, and lower levels of wellbeing. Feelings of loneliness did not appear to moderate the relationship between the perceived representation of obesity in media (i.e., perceived experiences of weight-related stigma) and mental wellbeing. We encourage greater media accountability when representing people with obesity to reduce the stigmatisation of this group.

## Supplementary information

Supplementary Materials

## Data Availability

The dataset analysed during the current study is publicly available on the Open Science Framework (10.17605/OSF.IO/VK3RC).
