# Supplementary material for: Perceived weight-related stigma, loneliness, and mental wellbeing during COVID-19 in people with obesity: A cross-sectional study from ten European countries
Source: Int J Obes (Lond). 2022 Sep 14;46(12):2120–7. doi: 10.1038/s41366-022-01220-1 (PMC9472193; doi:10.1038/s41366-022-01220-1)
Supplement: Supplementary file 1 — Supplementary Materials [file 41366_2022_1220_MOESM1_ESM.docx]

**Perceived weight-related stigma, loneliness, and mental wellbeing during COVID-19 in people with obesity: A cross-sectional study from ten European countries. [SUPPLEMENTARY MATERIALS].**

Full breakdown of participants removed during Qualtrics data quality checking:

- Scrubbed Responses (removal of identifiable information): n=584
- Screened Responses (screened accordingly to eligibility): n=2919
- Overquota Responses (participants who attempted to take the survey after the quota for their demographics had already been met): n= 6891
- Quality Fail Responses (participants who failed any quality checks in the survey): n= 1667
- Dropouts (participants who attempted the survey but did not complete it): n=4310

Specific question wording for perceived experiences of weight-related stigma:

1. Compared to before COVID-19, during peak exposure of the outbreak has representation of people with larger bodies changed on:
   1. TV
      1. Yes, more positive representation
      2. Yes, more negative representation
      3. No change
   2. Social media
      1. Yes, more positive representation
      2. Yes, more negative representation
      3. No change

There was some minor heteroscedasticity in the PHQ data so we re-analysed the data using robust standard errors. This did not influence the pattern of results (Tables S1-S3).

Table S1: Robust regression predicting anxiety (PHQ)

| **Predictors** | **Unstandardised coefficient** | **Robust Standard Error** | **95% Confidence Interval** | **p-value** |
| --- | --- | --- | --- | --- |
| Age in years | -0.02 | <0.01 | -0.02 – -0.01 | <0.001 |
| Shielding (‘Yes’, with ‘No’ as base) | 0.31 | 0.06 | 0.18 – 0.43 | <0.001 |
| Body mass index (BMI) | <0.01 | 0.01 | -0.01 – 0.01 | 0.377 |
| Total loneliness | 0.33 | 0.02 | 0.30 – 0.37 | <0.001 |
| Sex (‘Female’, with ‘Male’ as base) | 0.52 | 0.06 | 0.39 – 0.64 | <0.001 |
| Racial group (‘white’ as base) | -0.01 | 0.10 | -0.21 – 0.19 | 0.931 |
| SES | 0.01 | 0.02 | -0.02 – 0.04 | 0.461 |
| More negative representation on television (‘no change’ as base) | 0.21 | 0.11 | -0.01 – 0.43 | 0.065 |
| More positive representation on television (‘no change’ as base) | 0.27 | 0.10 | 0.07 – 0.47 | 0.007 |
| More negative representation on social media (‘no change’ as base) | 0.32 | 0.11 | 0.10 – 0.53 | 0.004 |
| More positive representation on social media (‘no change’ as base) | 0.17 | 0.100 | -0.03 – 0.36 | 0.097 |

Table S2: Robust regression predicting depression (PHQ)

| **Predictors** | **Unstandardised coefficient** | **Robust Standard Error** | **95% Confidence Interval** | **p-value** |
| --- | --- | --- | --- | --- |
| Age in years | -0.01 | <0.01 | -0.02 – -0.01 | <0.001 |
| Shielding (‘Yes’, with ‘No’ as base) | 0.27 | 0.07 | 0.14 –0.40 | <0.001 |
| Body mass index (BMI) | 0.01 | 0.01 | -0.01– 0.02 | 0.264 |
| Total loneliness | 0.43 | 0.02 | 0.40 – 0.47 | <0.001 |
| Sex (‘Female’, with ‘Male’ as base) | 0.24 | 0.06 | 0.111– 0.36 | <0.001 |
| Racial group (‘white’ as base) | <0.01 | .10 | -0.20 – 0.20 | 0.973 |
| SES | -0.03 | 0.02 | -0.60 – 0.01 | 0.078 |
| More negative representation on television (‘no change’ as base) | 0.12 | 0.12 | -0.11– 0.35 | 0.311 |
| More positive representation on television (‘no change’ as base) | 0.21 | 0.10 | 0.01– 0.41 | 0.044 |
| More negative representation on social media (‘no change’ as base) | 0.54 | 0.11 | 0.32 – 0.75 | <0.001 |
| More positive representation on social media (‘no change’ as base) | 0.20 | 0.10 | -0.01 – 0.40 | 0.052 |

Table S3: Robust regression predicting wellbeing (WHO-5)

| **Predictors** | **Unstandardised coefficient** | **Robust Standard Error** | **95% Confidence Interval** | **p-value** |
| --- | --- | --- | --- | --- |
| Age in years | 0.07 | 0.03 | 0.01 – 0.12 | 0.022 |
| Shielding (‘Yes’, with ‘No’ as base) | -1.20 | 0.87 | -2.91 – 0.51 | 0.170 |
| Body mass index (BMI) | -0.16 | 0.07 | -0.30 – -0.02 | 0.021 |
| Total loneliness | -6.53 | 0.23 | -6.99 – -6.08 | <0.001 |
| Sex (‘Female’, with ‘Male’ as base) | -7.22 | 0.83 | -8.85 – -5.59 | <0.001 |
| Racial group (‘white’ as base) | -2.22 | 1.36 | -4.88 – 0.44 | 0.102 |
| SES | 0.30 | 0.21 | -0.12 – 0.72 | 0.159 |
| More negative representation on television (‘no change’ as base) | 1.14 | 1.53 | -1.85 – 4.13 | 0.456 |
| More positive representation on television (‘no change’ as base) | 4.76 | 1.37 | 2.07 – 7.45 | 0.001 |
| More negative representation on social media (‘no change’ as base) | -3.68 | 1.46 | -6.54 – -0.82 | 0.012 |
| More positive representation on social media (‘no change’ as base) | 1.80 | 1.35 | -0.84 – 4.44 | 0.180 |

Table S4: Participants characteristics for key study outcomes by country of residence.

| **Characteristic (n=2882)**  Mean, SD (unless otherwise specified) | | **England**  **(n=287)** | **Greece**  **(n=297)** | **Sweden**  **(n=288)** | **Italy**  **(n=294)** | **France**  **(n=279)** | **Spain**  **(n=294)** | **Portugal**  **(n=295)** | **Israel**  **(n=290)** | **Denmark**  **(n=282)** | **Germany**  **(n=276)** |
| --- | --- | --- | --- | --- | --- | --- | --- | --- | --- | --- | --- |
| Total distress (PHQ) | | 5.16 (3.95) | 4.06 (2.96) | 4.10 (3.68) | 4.39 (3.65) | 3.88 (3.33) | 4.21 (3.51) | 4.63 (3.43) | 4.00 (3.17) | 3.06 (3.30) | 3.92 (3.38) |
| Anxiety (PHQ subscale) | | 2.54 (2.08) | 1.95 (1.68) | 1.90 (1.97) | 2.21 (1.93) | 1.87 (1.77) | 1.94 (1.82) | 2.33 (1.85) | 2.06 (1.75) | 1.31 (1.70) | 1.80 (1.79) |
| Depression (PHQ subscale) | | 2.62 (2.09) | 2.11 (1.56) | 2.20 (1.88) | 2.18 (1.95) | 2.01 (1.84) | 2.27 (1.92) | 2.31 (1.87) | 1.94 (1.70) | 1.74 (1.85) | 2.12 (1.83) |
| Wellbeing (WHO-5) | | 37.90 (24.32) | 43.10 (23.53) | 46.86 (23.09) | 45.99 (23.95) | 48.72 (25.15) | 48.18 (25.31) | 42.45 (22.82) | 45.19 (22.15) | 48.09 (24.29) | 45.09 (24.15) |
| Total loneliness (DJG) | | 3.75 (1.72) | 3.24 (1.88) | 3.37 (1.81) | 3.50 (1.91) | 3.41 (1.68) | 3.32 (1.85) | 3.64 (1.78) | 3.25 (1.69) | 2.99 (1.95) | 3.22 (1.78) |
| Representation of people with larger bodies on television during peak exposure of Covid-19, compared to before Covid-19 [n, %] | More negative | 70 (24.4) | 50 (16.8) | 56 (19.4) | 40 (13.6) | 52 (18.6) | 46 (15.6) | 45 (15.3) | 75 (25.9) | 41 (14.5) | 35 (12.7) |
|  | No change | 161 (56.1) | 180 (60.6) | 185 (64.2) | 181 (61.6) | 191 (68.5) | 167 (56.8) | 171 (58.0) | 176 (60.7) | 183 (64.9) | 188 (68.1) |
|  | More positive | 56 (19.5) | 67 (22.6) | 47 (16.3) | 73(24.8) | 36 (12.9) | 81 (27.6) | 79 (26.8) | 39 (13.4) | 58 (20.6) | 53 (19.2) |
| Representation of people with larger bodies on social media during peak exposure of Covid-19, compared to before Covid-19 [n, %] | More negative | 56 ( 19.5) | 45 (15.2) | 51 (17.7) | 38 (12.9) | 48 (17.2) | 36 (12.2) | 35 (11.9) | 64 (22.1) | 31 (11.0) | 33 (12.0) |
|  | No change | 172 ( 59.9) | 204 (68.7) | 199 (69.1) | 194 (66.0) | 188 (67.4) | 181 (61.6) | 197 (66.8) | 186 (64.1) | 206 (73.0) | 199 (72.1) |
|  | More positive | 59 ( 20.6) | 48 (16.2) | 38 (13.2) | 62 (21.1) | 43 (15.4) | 77 (26.2) | 63 (21.4) | 40 (13.8) | 45 (16.0) | 44 (15.9) |
| *Abbreviations: SD – Standard Deviation, PHQ – Patient Health Questionnaire, WHO-5 - World Health Organisation Five Wellbeing Index, DJG - De Jong Gierveld short scale.* | | | | | | | | | | | |

Table S5: Associations between explanatory variables and total distress (controlled from age, shielding, body mass index, total loneliness, and sex).

| **Representation of people with larger bodies during peak exposure of the outbreak, compared to before Covid-19** | | **Estimated association with total distress when compared to no change (Unstandardised coefficient, 95% CI)** |
| --- | --- | --- |
| Representation on television  (‘no change’ as base) | More negative representation | 0.27 (-0.13, 0.68) |
|  | More positive representation | 0.45 (0.09, 0.81) |
| Representation on social media  (‘no change’ as base) | More negative representation | 0.8 (0.42, 1.19) |
|  | More positive representation | 0.34 (-0.02, 0.70) |
| Proportion of total variance (Conditional R^2^) | | 0.27 |
| *Abbreviations: CI – Confidence Interval* | | |

Table S6: Effect of control variables on regression predicting anxiety

| **Predictors** | **Unstandardised coefficient** | **Robust Standard Error** | **95% Confidence Interval** | **p-value** |
| --- | --- | --- | --- | --- |
| Age in years | -0.02 | <0.01 | -0.02 – -0.02 | <0.001 |
| Shielding (‘Yes’, with ‘No’ as base) | 0.29 | 0.06 | 0.17 – 0.42 | <0.001 |
| Body mass index (BMI) | 0.01 | 0.01 | -0.01 – 0.02 | 0.295 |
| Total loneliness | 0.34 | 0.02 | 0.30 – 0.37 | <0.001 |
| Sex (‘Female’, with ‘Male’ as base) | 0.51 | 0.06 | 0.39 – 0.64 | <0.001 |
| Racial group (‘white’ as base) | -0.02 | 0.10 | -0.22 – 018 | .845 |
| SES | <0.01 | 0.02 | -0.03 – 0.03 | .953 |
| More negative representation on television (‘no change’ as base) | 0.19 | 0.11 | -0.04 – 0.41 | 0.100 |
| More positive representation on television (‘no change’ as base) | 0.26 | 0.10 | 0.06 – 0.46 | 0.011 |
| More negative representation on social media (‘no change’ as base) | 0.31 | 0.11 | 0.10 – 0.52 | 0.004 |
| More positive representation on social media (‘no change’ as base) | 0.17 | 0.10 | -0.03 – 0.36 | 0.093 |

Table S7: Effect of control variables on regression predicting depression

| **Predictors** | **Unstandardised coefficient** | **Robust Standard Error** | **95% Confidence Interval** | **p-value** |
| --- | --- | --- | --- | --- |
| Age in years | -0.01 | <0.01 | -0.02 – -0.01 | <0.001 |
| Shielding (‘Yes’, with ‘No’ as base) | 0.28 | 0.06 | 0.15 – 0.40 | <0.001 |
| Body mass index (BMI) | 0.01 | 0.01 | -0.01 – 0.01 | 0.389 |
| Total loneliness | 0.43 | 0.02 | 0.39 – 0.46 | <0.001 |
| Sex (‘Female’, with ‘Male’ as base) | 0.24 | 0.06 | 0.12 – 0.36 | <0.001 |
| Racial group (‘white’ as base) | -0.05 | 0.10 | -0.25– 0.14 | .602 |
| SES | -0.03 | 0.02 | -0.06 – -0.01 | .030 |
| More negative representation on television (‘no change’ as base) | 0.09 | 0.11 | -0.13 – 0.31 | 0.429 |
| More positive representation on television (‘no change’ as base) | 0.19 | 0.10 | -0.01 – 0.39 | 0.054 |
| More negative representation on social media (‘no change’ as base) | 0.50 | 0.11 | 0.29 – 0.71 | <0.001 |
| More positive representation on social media (‘no change’ as base) | 0.17 | 0.10 | -0.02 – 0.37 | 0.08 |

Table S8: Effect of control variables on regression predicting wellbeing

| **Predictors** | **Unstandardised coefficient** | **Robust Standard Error** | **95% Confidence Interval** | **p-value** |
| --- | --- | --- | --- | --- |
| Age in years | 0.05 | 0.03 | 0.01 – 0.10 | 0.049 |
| Shielding (‘Yes’, with ‘No’ as base) | -1.29 | 0.81 | -2.88 – 0.30 | 0.113 |
| Body mass index (BMI) | -0.14 | 0.06 | -0.27 – -0.01 | 0.032 |
| Total loneliness | -6.32 | 0.22 | -6.75 – -5.90 | <0.001 |
| Sex (‘Female’, with ‘Male’ as base) | -6.79 | 0.78 | -8.31 – -5.27 | <0.001 |
| Racial group (‘white’ as base) | -2.45 | 1.26 | -4.93– 0.02 | .052 |
| SES | 0.25 | 0.20 | -0.14 – 0.64 | .213 |
| More negative representation on television (‘no change’ as base) | 0.99 | 1.42 | -1.79 – 3.78 | 0.485 |
| More positive representation on television (‘no change’ as base) | 4.52 | 1.28 | 2.01 – 7.02 | <0.001 |
| More negative representation on social media (‘no change’ as base) | -3.52 | 1.36 | -6.19 – -0.86 | 0.01 |
| More positive representation on social media (‘no change’ as base) | 1.99 | 1.25 | -0.47 – 4.45 | 0.112 |
